# Knowledge and skills of emergency physicians in managing traumatic dental injuries

**DOI:** 10.1007/s00068-021-01808-8

**Published:** 2021-10-24

**Authors:** S. Wolfer, N. von Hahn, D. Sievers, Ch. Hohenstein, P. Kauffmann

**Affiliations:** 1 Department of Oral and Maxillofacial Surgery, University Medical Center Goettingen, Robert Koch Straße 40, 37075 Goettingen, Germany; 2 Emergency Department, Hospital Bad Berka, Robert-Koch-Allee 9, 99437 Bad Berka, Germany

**Keywords:** Traumatic dental injury, Emergency care, Knowledge, Skills

## Abstract

**Purpose:**

Emergency departments are frequently confronted with traumatic dental injuries (TDIs). The prognosis of the injured tooth is related to early dental trauma management. For this reason, physicians must be familiar with the appropriate management of TDI. This study aimed to investigate the knowledge and skills of German emergency physicians regarding TDI.

**Methods:**

An electronic questionnaire was sent to 438 emergency departments throughout Germany. Four hundred and twenty seven questionnaires were evaluated and included in the analysis. The survey contained questions about physician characteristics and assessed their knowledge and skills of managing dental trauma. For statistical analysis, the Kruskal–Wallis, Mann–Whitney *U *test or ANOVA test was used as appropriate. Rank correlations were performed with the Spearman’s rank correlation.

**Results:**

Out of 427 participants, 256 (59.95%) stated they had no or insufficient knowledge, and 266 (71.12%) stated they had no skills in dental trauma management. Almost 76% of the participants had no previous knowledge of dentistry. Only 7.28% knew the right procedure for replanting an avulsed tooth. Just 26.06% would choose the right medium for temporary tooth storage. Having a dentist in the family (*p* = 0.0074) or clinical exposure to patients with dental trauma (*p* = 0.0384) influenced the results of the knowledge score.

**Conclusion:**

The knowledge and skills in dental trauma management among German emergency physicians are generally inadequate. Targeted training courses are necessary to ensure early and adequate TDI treatment to reduce the resulting medical and societal costs as much as possible.

**Supplementary Information:**

The online version contains supplementary material available at 10.1007/s00068-021-01808-8.

## Introduction

Emergency departments (EDs) are frequently confronted with dental issues. Up to 66% of tooth-related ED visits are for managing traumatic dental injuries (TDIs) in different variations [1]. Approximately, one-third of primary teeth and one-fifth of permanent teeth suffer traumatic injury in their lifetime [2]. TDI is often more time-consuming and costly than many other accidental injuries presenting to the ED [3]. Furthermore, the prognosis of the injured tooth is related to the early beginning of correct dental trauma management [2, 4]. However, a dentist is not usually available in ED. Therefore, emergency physicians must be familiar with the appropriate management of TDI [5, 6] However, numerous studies report a low level of knowledge and insufficient skills of emergency physicians worldwide and in Europe [6–12]. Since 2017, oral and maxillofacial procedures have been newly included in the European Core Curriculum for Emergency Medicine [13]. This study aimed to investigate the particular knowledge and skills that German emergency physicians have regarding TDI.

## Materials and methods

The study was reported to the local ethics committee and classified as a survey study among medical professionals; therefore this study did not require an ethical review.

### Participants

In total, 438 hospitals in Germany were contacted via e-mail with the request to participate in that study. A cover letter, which explains the nature and purpose of the study and clearly emphasizes its voluntary nature, was sent together with a link to an electronic survey to the heads of each ED. The heads of departments carried the distribution of the link to their staff. The return of the survey implied the consent of the participants. Confidentiality was maintained, as the questionnaire did not require the names or contact information of the participants. For simplification, the participants in this study are named emergency physicians, although this specialty and correlated training does not yet exist in Germany. All participants were working in emergency medicine at the time of that study from May 1st to July 31st 2020.

### Survey

The survey was developed based on former studies [6, 9, 10, 12]. The electronic questionnaire was created by the program Kwiksurveys®, Bristol UK (www.kwiksurveys.com). It was designed that the participants could only fill out one questionnaire, and a double filling was excluded. The original prequestionnaire was first given to ten employees of the local ED to evaluate the comprehensibility of non-dental staff and final adjustments were made. The questionnaire has two parts. Part one asks personal information, such as age, gender, profession, academic title, position, level of experience, type of ED regarding the connection to dentistry or maxillofacial surgery, a degree in emergency medicine, self-assessment of own knowledge and skills in dental trauma management, previous dental knowledge, a dentist in the family, interest in learning dental trauma management, wanted training resources, the approximate number of TDI cases per month, preferred persons for support in TDI management and for management of one´s own TDI. Part two asks specific questions about managing avulsed, dislocated and fractured teeth. There were 13 questions with eight multiple-choice and five yes-or-no questions that counted for a knowledge score based on the study by Yigit [9]. Zero to five correctly answered questions were noted as insufficient, six to nine as moderate, and 10 to 13 correctly responded to questions as high level of knowledge. Some important questions were designed as dependent questions. The participant could only see the follow-up questions if the answer was correct. If the answer was wrong, the entire set of question complexes was rated as wrong. Thus, logical inferences about answers through follow-up questions were avoided. The following fields in knowledge were asked: "Should permanent/deciduous teeth be replanted, repositioned, treated?" "In what time interval from the accident should the replantation of the teeth take place?" “What is the correct tooth storage medium?” “Whether and within what time interval should the patient see a dentist after first aid care?".

The actual guidelines for managing dental trauma published by the International Association of Dental Traumatology were used [14–16].

### Statistical analysis

Statistical analysis was performed with GraphPad Prism 9 (San Diego, CA, USA). Descriptive analysis was performed and comprised the mean, median, standard deviation, minimum and maximum. The Kruskal–Wallis, Mann–Whitney *U* test or ANOVA test was used as appropriate. Rank correlations were performed with the Spearman’s rank correlation. The level of significance was set at *p* < 0.05. In the case of correlations, a correlation coefficient of [*r*] = 0.1 was interpreted as low, of [*r*] = 0.3 as moderate and of [*r*] = 0.5 as high [17].

## Results

In total, 516 questionnaires were received by the program Kwiksurveys®. Because of major incompleteness, 89 questionnaires were excluded leaving a final total of 427 emergency physicians who participated. The mean age of the participants was 41.83 ± 9.48 years (median 40 years). Nearly half of them were anesthetists with 48.40%. The others were surgeons (36.12%), physicians of internal medicine (11.79%), and others (3.96%) which included general medicine, ear-nose-and-throat medicine, pediatrics, and gynecology. Table 1 summarizes all personal data of the participants.

**Table 1:** Characteristics of participants

Variable	Number (%)
Age (Mean/Median [Min–Max] in years)	41.83 ± 9.48/40 [21–75]
Gender	
Male	292 (68.38)
Female	135 (31.62)
Academic degree	
Prof	11 (2.67)
PD	11 (2.67
Dr	224 (54.37)
Non	166 (40.29)
Profession	
Anesthesiology	197 (48.40)
Surgery	147 (36.12)
Internal medicine	48 (11.79)
Others	15 (3.96)
Position	
Resident	114 (26.76)
Specialist	115 (27.0)
Consultant	131 (30.75)
Head of department	66 (15.49)
Year of training (Residents)	
1	8 (7.34)
2	10 (9.17)
3	21 (19.27)
4	23 (21.0)
5	23 (21.0)
6	19 (17.43)
> 6	5 (4.59)
Experience in ED (Mean/Median [Min–Max]in month)	97.42 ± 104.6/60 [0–504]
Amount of exposure to TDI per month	
0	245 (58.3)
1–10	134 (34.5)
11–20	12 (2.9)
> 20	0 (0)
Emergency work mostly in	
Preclinic	202 (49.63)
ED with OMFS	0 (0.0)
ED with Dentist	60 (14.74)
ED with OMFS & dentist	36 (8.85)
ED without OMFS & dentist	109 (26.78)
Degree in emergency medicine	
Non	43 (10.34)
Non but strive for it	34 (8.17)
Yes within common trunk in surgery	13 (3.13)
Yes, additional designation EM	281 (67.55)
Yes, additional training EM	42 (10.10)
Foreign degree EM	3 (0.72)
Family member as a dentist	
Yes	37 (8.9)
No	381 (91.1)
Previous dental knowledge	
Non	319 (75.59)
Yes, from studies	75 (17.77)
Yes, self-acquired	28 (6.64)

*ED* emergency department, *TDI* traumatic dental injury, *Prof* professor, *PD* participant with habilitation (German: “Privatdozent”), *Dr* doctoral degree in medicine, *OMFS* oral and maxillofacial surgeon, *EM* emergency medicine

The self-confidence to manage dental trauma was poor. The participants stated in 59.95% that they had no or insufficient knowledge and in 71.12% that they had no skills. Almost 76% indicated that they had no previous knowledge in dentistry. Only 7.28% knew the right procedure for replanting an avulsed tooth. Just 26.06% would choose a physiological nutrient medium for temporary tooth storage; only 11.22% selected milk. (Tables 2, 3 and 4).

**Table 2:** Questions about dental trauma management

Question	Answer	Number (%)
Do you have knowledge about dental trauma management? (Multiple choices possible)	No	177 (34.8)
	Yes, from books	104 (20.5)
	Yes, from colleagues	50 (9.8)
	Yes, from advanced training	68 (13.4)
	Yes, from clinical experience	96 (18.9)
	Yes, from other colleagues	13 (2.6)
How do you rate your knowledge in dental trauma management? (self-assessment)	Very good	0 (0)
	Good	13 (3.04)
	Sufficient	158 (37)
	Inadequate	205 (48.01)
	Have no knowledge	51 (11.94)
How do you rate your practical skills of dental trauma management? (Self-assessment)	Can do it by myself, without instructions	27 (7.22)
	Can do it by myself, with instructions	81 (21.66)
	Have no practical skills	266 (71.12)
Are you interested in learning dental trauma management?	Yes	231 (54.48)
	No	58 (13.68)
	Maybe	135 (31.84)
How should the knowledge be conveyed?	Books	7 (1.67)
	Video Demonstration	150 (35.71)
	Oral Training	48 (11.43)
	Hands on Courses	215 (51.19)

Questions were asked about the knowledge, the self-assessment of knowledge and skills in dental trauma management, the interest in learning dental trauma management, and how new knowledge and skills should be presented

**Table 3:** Distribution of responses regarding ideal dental storage media

Storage media	Number	(%)
Hypotone saline solution	1	0.33
Hypertone saline solution	0	0
Isotone saline solution	139	(45.87)
Saliva	18	(5.94)
Milk	**34**	**(11.22)**
Special nutrient medium	**82**	**(27.06)**
In a bag	8	(2.64)
In a dry cloth, gauze	3	1.0
No matter	1	0.3
I don´t know	17	(5.61)

Bold numbers are the statistical significant variables with *p* < 0.05

**Table 4:** Questions about specific dental trauma management for a) avulsed, b) dislocated, and c) fractured teeth

Question	Answer	a)		b)		c)	
	%	*n*	%	*n*	%	*n*	%
Should a permanent a) avulsed, b) luxated tooth be a) replanted, b) repositioned?	Yes	303	71.0	312	73.4	–	–
	No	13	3.0	7	1.7	–	–
	I don´t know!	111	26.0	91	21.4	–	–
When should a permanent avulsed tooth be a) replanted?	immediately	208	69.10	–	–	–	–
	Up to 6 h	49	16.28	–	–	–	–
	Up to 12 h	13	4.32	–	–	–	–
	Up to 24 h	18	5.98	–	–	–	–
	No matter	0	0.0	–	–	–	–
	I don´t know!	13	4.32	–	–	–	–
Do you know the procedure for a) replantation b) reposition? c) Do you know the classification of fractured teeth?	Yes	22	7.28	9	2.9	20	4.84
	No	246	81.45	211	67.2	393	95.16
	I am not sure	34	11.26	94	29.9	–	–
Should a patient be referred to a dentist after an emergency a) replantation, b) repositioning, c) first aid for fractured teeth?	Yes	295	97.68	300	96.2	379	91.8
	No	1	0.3	2	0.6	4	1.0
	Maybe	6	2.0	10	3.2	30	7.3
When should the presentation take place at the dentist after an emergency a) replantation, b) repositioning, c) first aid for fractured teeth?	Immediately	41	13.90	43	14.2	63	16.4
	Up to 24 h	135	45.76	147	48.7	183	47.5
	Up to 1 week	74	25.08	71	23.5	85	22.1
	On demand	0	0.0	1	0.3	6	1.6
	I don´t know	45	15.25	40	13.3	48	12.5

*h* hours, *n* number

The second part of the questionnaire evaluates special knowledge about dental trauma management with 13 questions summarized in a knowledge score. The mean score was 6.57 ± 2.41 (Median 7). More than half of the participants (52.46%) reached an average score, but only 16.86% had a high knowledge score. Only 2/450 participants had 13/13 correct answers. Table 5 shows the scores for different categories.

**Table 5:** Knowledge score for different categories

Parameter	*n*	Knowledge score (Median)	*p* value
Gender			
Male	292	6.61 (7.0)	0.5394
Female	135	6.41 (7.0)	
Academic degree			
Non	166	6.47 (7.0)	0.6840
Dr	224	6.71 (7.0)	
PD. Dr	11	6.0 (6.0)	
Prof. Dr	11	5.55 (7.0)	
Position			
Resident	114	6.04 (6.5)	0.0906
Specialist	114	6.84 (7.0)	
Consultant	131	6.53 (7.0)	
Head of department	66	6.97 (7.0)	
Profession			
Anesthesiology	197	6.16 (7.0)	**0.0157**
Surgery	147	7.14 (7.0)	
Internal medicine	48	6.33 (7.0)	
Others	15	7.0 (9.0)	
Degree emergency medicine			
No	77	6.07 (6.0)	0.3237
Yes	339	6.67 (7.0)	
Experience in EM			
Preclinical	202	6.31 (7.0)	**0.0186**
Clinical	205	6.96 (7.0)	
Amount of exposure to TDI per month			
0	245	6.28 (7.0)	**0.0384**
1–10	145	7.05 (7.0)	
10–20	12	6.92 (7.0)	
Dentist in family			
No	281	6.44 (7.0)	**0.0074**
Yes	27	7.70 (8.0)	
Previous dental knowledge			
Non	319	6.32 (7.0)	**0.0240**
From studies	75	6.96 (7.0)	
Self-acquired	28	7.82 (8.0)	

Bold numbers are the statistical significant variables with *p* < 0.05

*EM* emergency medicine, *TDI* traumatic dental injury, *Prof* professor, *PD* participant with habilitation (German: “Privatdozent”), *Dr* doctoral degree in medicine, *n* number

**Fig. 1:**
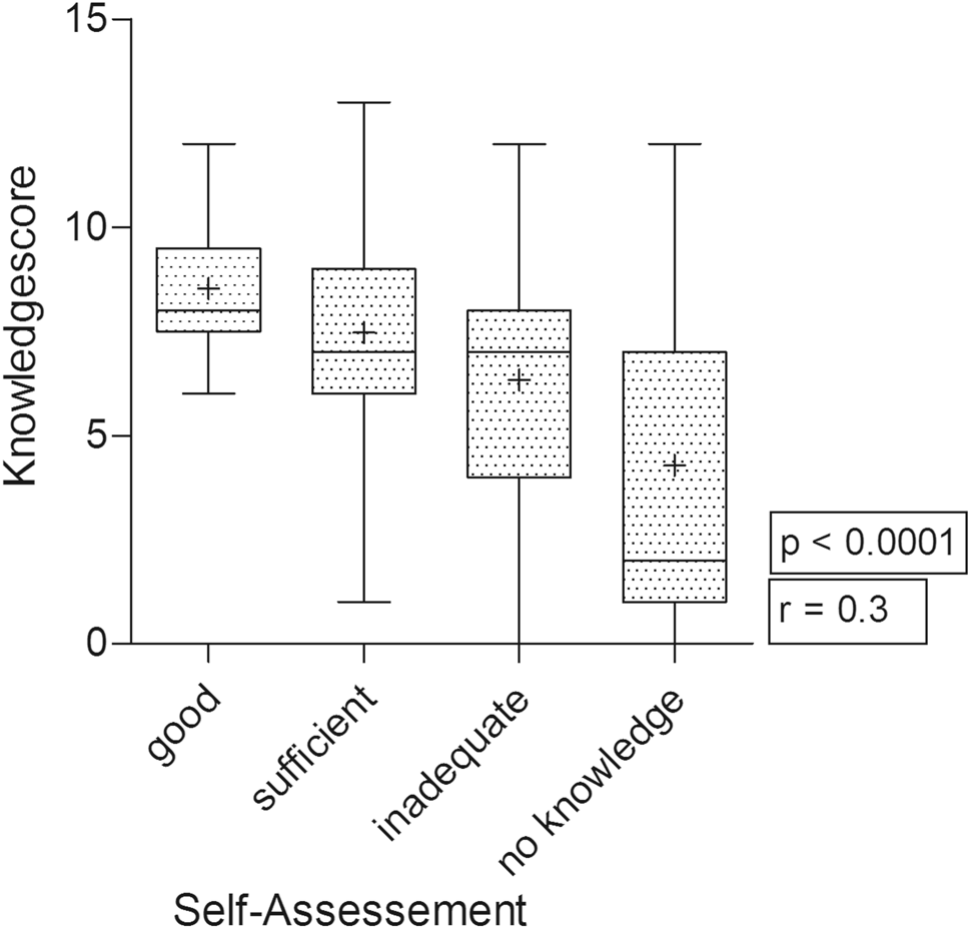
Self-assessment of knowledge and skills compared with the knowledge score. The knowledge score decreases with the self-assessment. *p* < 0.0001, Spearman’s correlation coefficient *r* = 0.3

The self-assessment of knowledge and skills was compared with the knowledge score (*p* < 0.0001). There was a correlation between them with a differential coefficient of *r* = 0.3. In that way, the results of the self-assessment can be concluded reliable (Fig. 1).

In general, there is a great willingness to learn TDI management correctly. Fifty-four percent of the participants wanted to have advanced training in dental trauma management. Just 13.68% do not want any training. They would like to have hands-on courses (51.19%) and video demonstrations (35.71%) for education. (Table 2) If they wished for support in the ED, 81.75% would like an oral and maxillofacial surgeon (OMFS), 63.75% would like hands-on help and 18% would like it by telephone. Approximately 17% wanted support from a dentist, with 13.38% hand-on help and 3.65% by telephone. Only two participants had enough book help, and 3 participants did not need help because they could manage dental trauma by themselves. The participants were asked to indicate who should treat their own TDI. Eighty-two percent wanted an OMFS, 14.99% wanted a dentist, and for just seven participants (1.64%), they would either go to an emergency specialist or it does not matter who treats their own TDI.

## Discussion

This survey found that the knowledge and skills of German emergency physicians were poor with 75.59% of the participants without previous dental knowledge and 59.95% with inadequate or no knowledge about TDI. Only 16.86% of the participants reached a high knowledge score, and 71.12% stated they had no specific skills in handling TDI. This confirms results from other previous studies all over the world summarized by Yeng 2019 [7]. In the current investigation, for avulsed teeth as the worst dental trauma, the general knowledge of the German participants was moderate, but the confidence to manage an avulsed tooth correctly was even worse. Approximately 70% would consider replantation for an avulsed tooth, 69% would replant an avulsed tooth immediately, 97.68% would send the patient after emergency replantation to a dentist and 59.66% would do that within 24 h after trauma. However, 81.45% of the participants had no idea how to replant the avulsed tooth. Similar results were shown by Bahammam where nearly 79.5% of emergency physicians considered replantation, but only 27.9% would do it by themselves [11]. Furthermore, more studies report poor knowledge of emergency physicians in managing TDI, particularly in managing avulsed teeth [5, 6, 8, 12]. The treatment of avulsed teeth has been recommended by the International Association of Dental Traumatology with proper storage and preservation of the tooth until it could be replanted, immediate replantation if possible, and splinting and follow-up with endodontic therapy by a dentist [14].

The choice of the right transport medium in our study was correct only in 27.06%, even including the second-best solution, milk, the number only increases to 38.28%. Conversely, this means that 61.72% of the avulsed teeth would be incorrectly stored from the beginning, and the tooth prognosis would worsen. Similar results were shown by Bahammam in 2018 [11].

As in a previous study [6], most of our participants (45.87%) chose the wrong medium isotonic saline solution. However, in the study by Aren, only moderately effective media were listed, and no physiologic nutrient medium as a selection choice existed. Therefore, the specified 94% correct answers of the transport medium must be assessed very critically.

A publication to emergency physicians regarding the correct tooth storage medium was published in 1990 to improve this situation [18]. In 2011, a publication was brought out especially for German physicians, which clearly stated a special nutrition solution, provided in special tooth lock boxes, as the best storage medium. They emphasized the importance of the correct and timely storage of an avulsed tooth [19]. The physiological nutrient medium contains the correct pH and electrolytes and keeps the periodontal ligament cells up to 24 h alive. If not available, cold UHT milk would be the second-best choice [18, 19]. Sterile saline lacks metabolically essential ions and does not provide glucose to the cells and damages the tooth cells in a short time [18, 19]. However, during this actual study, it was found that even after more than 30 years and after more publications on the subject over time, there is still a clear gap in knowledge about the storage medium. Since the prognosis depends directly on the time of the replantation, the choice of the storage medium is more important [4, 8, 11, 20] because the knowledge and self-confidence about replantation are as bad as reported above.

The tooth should be replanted immediately or up to 15 min after the accident [4, 14]. In this way, there is a chance of up to 90% retaining the tooth for life and reducing complications caused by delayed and incorrect treatment and saving costs for avoidable follow-up treatments [2, 18, 19, 21]. Thus, the tooth should at least be stored so that it can be adequately replanted by a dentist or maxillofacial specialist after a certain period. Under no circumstances should a tooth be transported dry or in tap water [18, 19].

The synopsis of the results also suggests that an interest or a connection with dental trauma management led to better results within this questionnaire. The participants having a dentist as a family member achieved a better knowledge score than those without. Yigit also describes the ease of consultation about TDI from having a dentist in the family as related to better knowledge [9]. Similar findings were reported by another study, who found better knowledge when participants were married to a dentist [8].

The influence of the specialty and the experience level are controversial in the literature [5, 6, 8–12]. In two different studies, however, it was pointed out that disciplines with more exposure to TDI achieved better results [10, 12].

This is in line with our results, in which the participants who reported approximately 1–10 cases per month achieved better results than those without dental trauma cases. The pure time worked in the ED did not show any influence on knowledge in our study. For this reason, in the literature, one should look at what is meant by the term experience level.

The knowledge from self-study yielded better values, suggesting that those interested in the topic and those who have to deal with TDI cases receive further training more effectively than pure knowledge imparted from the course or without training. Special training of the TDI and the exposure to the TDI were also associated with better results in a previous study [10].

Interest in learning more about the management of TDI was expressed strongly in our study. Most of the German participants wanted practical courses followed by video demonstrations. This also shows that it is less general knowledge than imparting the right skills that need to be addressed. This is reported in several studies [6, 8, 9, 11, 12].

The choice of the desired attending physician in the event of one's own TDI is also an indication of poor self-confidence in the physician´s ability to deal with TDI. In our German study, only 1.64% would like to be treated by an emergency physician, and most would like an OMFS followed by a dentist. This fact was also seen by Trivedy et al. with similar results [12].

The connection of the ED participating in our study directly to OMFS or dentistry was only given to approximately 24% which means 76% of the participants work without a direct connection to a dental trained colleague. Other studies also describe similar things, but no better knowledge was proven if there was a connection to OMFS and/or dentistry [8, 10]. This aspect was not further specifically examined in our study.

German emergency physicians also largely wanted support in managing TDI; most of them wanted personal help from OMFS followed by an occasion of a telephone consultation or subsequently from dentists in the same way. Because our department of OMFS initiated that study, the preference for help could be biased in the case of the most expected answer.

A list of dental colleagues willing to advise emergency physicians on how to deal with dental trauma correctly, preferably 24/7 via telephone, was already suggested in a previous study [10] and is certainly regarded as useful. The mere advice of the correct transport medium can dramatically improve the prognosis of the injured tooth.

In addition, TDI should also be reflected in the training of medical professionals, at least in the training of emergency physicians. It was previously shown that education on a correct diagnosis of the TDI and more information about the importance of early management paired with the right skills could lead to a better outcome of the injured teeth [7]. Furthermore, this training should be recurring at best to maintain a good level of competency. It should contain facts about basic dental anatomy for trauma assessment and management, information about FDI (Fèdèration Dentaire Internationale) tooth numbering system for better communication with the dentist, basic principles of different types of TDI and theory and practical lessons about repositioning and replantation of teeth [7] For first aid, emergency physicians should realize the TDI, manage the patient´s pain and refer them to a dentist with sole tooth structure problems; with bone involvement they should refer them to an OMFS department. In case of luxation or avulsion, they should reposition or replant a permanent tooth as soon as possible and refer them to a dentist as soon as possible. Therefore, they should be familiar with the skills for that [7]; otherwise, they should know the right storage medium in case of an avulsed tooth.

Unfortunately, the management of TDI is not generally included in medical books or first aid textbooks and manuals [22, 23]. To solve the shortage of information about TDI management for European emergency physicians, since 2017, due to the new European Core Curriculum for Emergency Medicine, the new chapter “Oral and Maxillofacial Procedures” has been established [13]. Based on the new core curriculum, a new German book on clinical emergency medicine was published in 2020. It contains the theory and instructions on the practical procedure for dental trauma [24]. The effects of this additional information remain to be seen in the future.

In the ED, guidelines, and aids, such as the “Dental Trauma Guide” (www.dentaltraumaguide.org) or the special App “AcciDent®”, should be available. The suggestion to provide helpful TDI guidelines in the ED was already made before [10], which is certainly a sensible option.

Patients presenting to an ED due to TDI seek the best possible help. Many people, including teachers, are not self-confident enough to immediately treat an avulsed tooth, and fewer know the right storage media. They believe that the presentation in ED is the best choice after TDI [11, 20].

The current deficiency in knowledge and the inadequate skills of emergency physicians in the management of TDI needs to be improved for the patient´s benefit through targeted discussion of the topic and through training. This will benefit the patient and the society as a whole economically [25].

There are several limitations we have to point out in this study. First, 427 participants were a limited number compared to all emergency physicians in Germany. The results presented here may not completely reflect the situation that all emergency physicians would have been indicated. However, the participants came from all over Germany, and to our knowledge, this study evaluated the largest number of participants in such a study in comparison to all similar studies worldwide and can therefore be assumed to be representative. Second, the participation in this study was voluntary, so probably only those interested in the topic took part. The results may therefore have been influenced. Third, there was no control when completing the questionnaires. Participants could also have used books or the opinions of colleagues, friends, or relatives to answer the questions and thus influence the results.

Therefore, we conclude that the knowledge and skills in dental trauma management among German emergency physicians are generally inadequate. Targeted training courses are necessary to ensure early and adequate treatment of TDI and to be able to reduce the resulting medical and societal costs as much as possible.

## Supplementary Information

Below is the link to the electronic supplementary material.

Supplementary file1 (PDF 84 KB)

## Data Availability

Only anonymous data were collected and used. Upon special request, the data can be made available after the planned use has been checked.
