# Supplementary material for: Knowledge and skills of emergency physicians in managing traumatic dental injuries
Source: Eur J Trauma Emerg Surg. 2021 Oct 24;48(3):2081–8. doi: 10.1007/s00068-021-01808-8 (PMC9192501; doi:10.1007/s00068-021-01808-8)
Supplement: Supplementary file 1 — Supplementary file1 (PDF 84 KB) [file 68_2021_1808_MOESM1_ESM.pdf]

Editing Certificate

This document certifies that the manuscript

Knowledge and Skills of Emergency Physicians in Managing Traumatic Dental Injuries

prepared by the authors

Wolfer S, von Hahn N, Sievers D, Hohenstein Ch, Kauffmann P

was edited for proper English language, grammar, punctuation, spelling, and overall style by one or more of the highly qualified native English speaking editors at AJE.

This certificate was issued on **September 13, 2021** and may be verified on the AJE website using the verification code **D600-826F-55EB-3A7C-EC48**.

Neither the research content nor the authors' intentions were altered in any way during the editing process. Documents receiving this certification should be English-ready for publication; however, the author has the ability to accept or reject our suggestions and changes. To verify the final AJE edited version, please visit our verification page at aje.com/certificate. If you have any questions or concerns about this edited document, please contact AJE at support@aje.com.

AJE provides a range of editing, translation, and manuscript services for researchers and publishers around the world. For more information about our company, services, and partner discounts, please visit aje.com.
